# Effect of Graphene Aerosol Doped with Hypochlorous Acid, Curcumin, and Silver Nanoparticles on Selected Structural and Biological Properties

**DOI:** 10.3390/ma17225640

**Published:** 2024-11-18

**Authors:** Aleksandra Sowińska, Agata Lange, Marta Kutwin, Sławomir Jaworski, Wojciech Skrzeczanowski, Aneta Bombalska, Anna Romiszewska, Klaudia Olkowicz, Zdzisław Bogdanowicz, Barbara Nasiłowska

**Affiliations:** 1 Faculty of Mechanical Engineering, Military University of Technology, gen. S. Kaliskiego 2, 00-908 Warsaw, Poland; 2 Department of Nanobiotechnology, Institute of Biology, Warsaw University of Life Sciences, Ciszewskiego 8, 02-786 Warsaw, Poland; 3 Institute of Optoelectronics, Military University of Technology, gen. S. Kaliskiego 2, 00-908 Warsaw, Poland; 4 Aircraft Airworthiness Division, Air Force Institute of Technology, 01-494 Warsaw, Poland

**Keywords:** aerosol, graphene oxide, curcumin, silver nanoparticles, hypochlorous acid

## Abstract

This paper presents the results of studies on the effects of four types of aerosols containing an aqueous dispersed suspension of graphene oxide (GO) and an aqueous dispersed suspension of graphene oxide with the addition of curcumin (GO + C), silver nanoparticles (GO + Ag), and hypochlorous acid (GO + HClO) on selected structural and biological properties. Structural studies were carried out using electron microscopy, including a scanning electron microscope (SEM), scanning transmission electron microscopy (STEM), laser emission spectroscopy (LIBS), and absorption spectra in the infrared range attuned total reflectance (FTIR-ATR). The growth inhibition zone and viability of *Staphylococcus aureus* and *Pseudomonas aeruginosa* bacteria were studied. Studies have shown that the addition of silver nanoparticles and hypochlorous acid to the nanostructures of graphene oxide suspension improves bactericidal properties. In addition, it was observed that the application of a dispersed graphene oxide suspension in the form of an aerosol enriched with hypochlorous acid and silver nanoparticles results in the formation of a fairly uniform layer of graphene flakes, characterized by the presence of admixtures used.

## 1. Introduction

Since Andre Geim and Konstantin Novoselov won the Nobel Prize in 2010 [1,2], graphene and its derivatives aroused interest in many areas of science, such as chemistry, physics, materials science, and nanotechnology. 

Graphene and its derivatives, including graphene oxide (GO), reduced graphene oxide (rGO), and graphene quantum dots (GQD), have been widely studied as some of the most promising biomaterials for biomedical applications due to their unique properties, two-dimensional structure, large surface area, chemical and mechanical stability, good conductivity, as well as good biocompatibility [3,4,5,6,7,8,9,10,11,12,13,14]. In biomedicine, graphene has also been used in imaging and delivery of anti-cancer drugs [15].

In recent years, graphene-based applications included the possibility of using graphene-based materials as antibacterial agents [16] or carriers of gradually releasing drug platforms [17]. The conductivity of graphene oxide decreases with the increase in the number of oxygen groups. Depending on the production methods and substrates used, graphene oxide (GO) may exhibit antibacterial or bacteriostatic activity against Gram-positive and Gram-negative bacteria. However, this is mainly related to the physical and chemical interactions between graphene oxide flakes and bacterial cells [17,18,19]. According to [20], the edges of graphene nanoparticles can cut through the bacterial cell wall and membrane, allowing the leakage of the intracellular matrix, which ultimately leads to cell death. In addition, it is believed that the antibacterial activity of GO may also result from oxidative stress caused by the overproduction of reactive oxygen species (ROS) [21,22]. 

Graphene and its derivatives provide a suitable platform for the development of nanocomposites that allow the combination of different molecules or nanomaterials that result in providing new materials with improved or new functionality [17].

Such materials include curcumin (diferuloylmethane), which is a natural polyphenol. In addition to its culinary use as a flavoring and food colorant, curcumin also has antioxidant, anti-inflammatory, antimicrobial, and anticancer properties. However, it exhibits poor solubility and bioavailability [23,24]. Curcumin works by forming transmembrane pores or ion channels in the bacterial cell membrane. These porous structures lead to the leakage of metabolites that are essential for the proper functioning of the bacterial cell. As a result of the formation of these microchannels, the structure of the bacterial cell wall is destabilized. The cell wall, which is the basic protective barrier of bacteria, loses its integrity, thus preventing the proper flow of nutrients and other molecules necessary for the synthesis of new cells. As a result, metabolic processes in the cell are disrupted, which can lead to inhibition of its growth, and eventually to the death of the bacterial cell. Curcumin is also believed to have the ability to directly affect various cellular components, e.g., it can bind to proteins and lipids in the cell membrane, which contributes to further violation of the structure and function of this membrane. In addition, studies presented by Rai et al. [25] have shown that curcumin induces oxidative stress in bacterial cells, which in turn leads to DNA, protein, and lipid damage. The results of studies on the effects of curcumin as a bactericidal agent were confirmed by a team led by Margherita Cacaci [26], which also demonstrated the possibility of combining graphene oxide nanoparticles with curcumin (GO/CU).

The synthesis of graphene composites with various metal nanoparticles due to their antibacterial activity is also interesting [27]. A relatively well-known nanocomposite with antibacterial and anticancer properties is the combination of graphene with silver nanoparticles, which significantly inhibits the development of pathogens. The combination of silver with graphene particles, graphene oxide, and reduced graphene oxide clearly enhances the antibacterial activity [28]. 

The results of the study [29] show that the nanostructures of graphene oxide with silver and reduced graphene oxide with silver have antibacterial activity against *E. coli* and *S. aureus* bacteria. Reduced graphene oxide in this case showed a much better effect on inhibiting bacterial growth. In addition, the results obtained in these studies indicate that rGO/AgNP can potentially be used as adjuvant agents to improve the therapeutic effect of chemotherapy, especially when platinum-based. 

The silver nanoparticles interaction mechanism involves passing through the cell membrane and binding to various intracellular components, such as proteins and nucleic acids. Silver’s binding to enzymatic proteins leads to their denaturation and loss of function, which prevents bacteria from carrying out key biochemical reactions. The interaction of silver with bacterial DNA can cause disorders in the processes of replication and transcription, resulting in the inhibition of bacterial cell growth and division.

Another interesting substance with antibacterial and antifungal activity is hypochlorous acid [30,31], which not only affects the bacterial cell membrane, leading to its destabilization, but also destroys the internal structures of the cell, oxidizing key macromolecules, including proteins, enzymes, lipids, and nucleic acids, which in turn leads to cell death. The biocidal effect of HClO is the oxidation of cell building blocks, i.e., proteins, lipids, and nucleic acids. In the case of bacteria, membrane proteins and enzymes are oxidized, which leads to disruption of metabolic processes.

Currently, there are many applications of graphene oxide as a platform for transferring nanoparticles [28]. However, it is mainly used in powder form, applied as a suspension [29], or used as a pre-prepared dry medium [30]. Graphene aerosol used without admixtures and other supporting substances was the subject of research in the publication [32]. It has been shown that the deposition of a graphene oxide layer in the form of an aerosol causes a decrease in the viability of cancer cells. This publication supplements the current state of knowledge by presenting the effect of dispersed graphene oxide aqueous suspension with additions of curcumin, silver nanoparticles, or hypochlorous acid applied in the form of an aerosol to selected Gram-negative and Gram-positive bacteria.

## 2. Materials and Methods

### 2.1. Method of Making Graphene Aerosol

A dispersed suspension of graphene oxide 4.5 g/L produced and purchased from the Łukasiewicz Research Network—Institute of Electronic Materials Technology (Warsaw, Poland) was used to carry out the research. This suspension was mixed with curcumin, silver nanoparticles (NANO-TECH Polska, Warsaw, Poland), and hypochlorous acid at a concentration of 500 ppm (BioMedAqua Sp. z o.o., Dębica, Poland) according to Table 1.

The substances included in the aerosols were thoroughly combined using a magnetic stirrer (10 min, temp. 21 °C, 500 ppm, Heidolph Scientific Products GmbH, Schwabach, Germany) to obtain a homogeneous mixture that can be easily applied from a container in the form of an aerosol. The curcumin was previously sifted through a stainless steel sieve with a mesh size of ~50–60 μm, and then 0.1 g was dissolved in 100 mL of distilled water using a Vortex stirrer (10,000 rpm, temp. 22 °C, BioSan, Riga, Latvia). The mixture prepared in this way was added and combined with a suspension of 500 mL of graphene oxide using a magnetic stirrer (10 min, temp. 21 °C, 500 ppm, Heidolph Scientific Products GmbH, Schwabach, Germany). 

**Figure 1:**
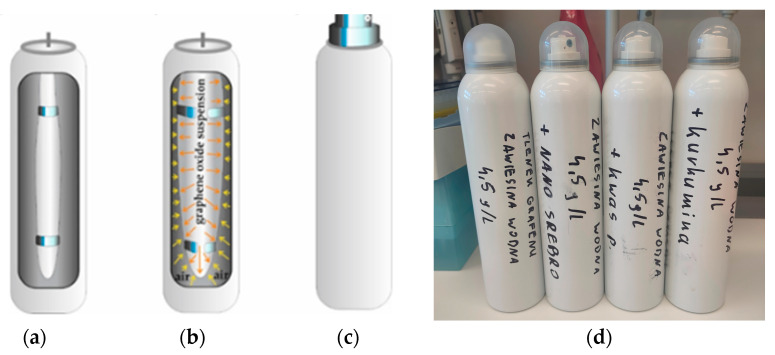
Diagram of aerosol production using the bag on valve method, (**a**) placing the bag in a container, (**b**) filling the bag with the selected mixture so that excess pressure is created in the space between the bag and the inside of the housing, (**c**) diagram of the finished product, and (**d**) photo of the finished GO aerosols, GO + C, GO + Ag, and GO + HClO intended for research.

GO, GO + C, GO + Ag, and GO + HClO aerosols were made using the bag on valve method, which involves placing a bag with the substance inside the container and filling the space between the bag and the container with a non-flammable gas, such as atmospheric air or nitrogen, under pressure. A valve was attached to the bag, which, when pressed, caused the release of the substance in the form of an aerosol due to the excess air pressure between the bag with the substance and the aerosol housing (Figure 1).

### 2.2. Research Methods

#### 2.2.1. Surface Morphology

- Scanning electron microscope—SEM.
  The surface morphology of the graphene paper was determined using scanning electron microscopy (SEM) (Quanta 250 FEG SEM, FEI, Hillsboro, OR, USA). A SEM image was created with a distributed detector (ETD-BSE, FEI, Hillsboro, OR, USA) with an acceleration voltage of 5 kV for GO and 10 kV.
  The SEM image of GO, GO + C, GO + Ag, and GO + HClO aerosols were taken after their application on the surface of a glass slide.
- Scanning transmission electron microscopy—STEM.
  To take the STEM images (Quanta 250 FEG SEM, FEI, Hillsboro, OR, USA), a copper TEM mesh was used, on which a layer of tested substances (GO, GO + C, GO + Ag, and GO + HClO) in the form of aerosols was applied.
- Fourier transform infrared spectroscopy (FTIR) study of the chemical surface composition.
  Aerosol GO, GO + C, GO + Ag, and GO + HClO were analyzed by FTIR (Nicolet IS50, FTIR, ThermoFisher SCIENTIFIC, Waltham, MA, USA). Using ATR mode in a range of 400–4000 cm^−1^ with a resolution of 4 cm^−1^ and 64 scans [16].
- Laser-induced breakdown spectroscopy—LIBS.
  Research on this was carried out in the experimental setup presented in the publication [33]. The plasma was generated using a pulsed Nd:YAG laser, Brio model, by Quantel, wavelength 1064 nm, pulse duration 4 ns. The radiation emitted by the plasma was recorded with a spectrometer using an optical head and optical fiber.
- Contact angle.
  The contact angle was measured using an optical microscope (6000 VHX, Keyence Corporation, Osaka, Japan). Droplets of ultrapure water with a volume of about 3 µL from a constant height of 5 mm were dropped onto the surface of a laboratory slide with GO, GO + C, GO + Ag, and GO + HClO aerosol applied 24 h earlier.
- The ζ-potential.
  The ζ-potential of GO + C, GO + Ag, and GO + HClO were measured using a Zetasizer ZSP (Malvern Instruments Ltd., Malvern, UK) at 25 °C based on laser Doppler velocimetry techniques. Before measurement, the aerosol was suspended in ultrapure water and homogenized using an ultrasonication probe for 30 min. The ζ-potentials of the aerosols were measured using the laser dynamic scattering electrophoretic method, applying the Smoluchowski approximation with a Zetasizer Nano ZS90 (Malvern Instruments, Malvern, UK). Each sample was measured after stabilizing at 25 °C for 120 s. All measurements were performed in triplicate.

#### 2.2.2. Bacteriological Experiments

- Bacterial growth inhibition zone
  *Staphylococcus aureus* (ATCC 25923) and *Pseudomonas aeruginosa* (ATCC 27853) were obtained from the American Type Culture Collection (ATCC) in the form of spore suspension, and bacterial strains were maintained in 20% (*v*/*v*) glycerol at −20 °C. Before use in experiments, glycerol was removed by washing with distilled water. Bacterial strains were cultured in tryptic soy agar (TSA) in standard conditions (24 h, 37 °C). A total of 10 mL of nutrient agar (BioMaxima, Lublin, Poland) was placed on Petri dishes (90 mm in diameter). Then, pour plating was performed onto nutrient agar with appropriate bacterial suspension (1.5 × 10^8^ cells/mL). In order to create an area with a diameter of 2.7 cm, the application of the aerosol was carried out through bushing with a diameter of 2.7 cm and a length of 5 cm. These areas (for *S. aureus* and *P. aeruginosa*) were applied to the solidified agar and plates were incubated for 24 h at 37 °C. Results were determined by the zone of growth inhibition.
  The bacterial strains used in the study result from the recommendations in the ISO 20645:2004 standard [34], which specifies a method for the determination of the effect of antibacterial treatments applied to flat textiles. They have been selected on the basis of a literature review, which indicates that *Staphylococcus aureus* and *Pseudomonas aeruginosa* infections are among the most common etiogenic agents in nosocomial infections [16].
- Bacterial viability
  In order to check the viability of bacteria after the application of GO, GO + C, GO + Ag, and GO + HClO aerosols, samples were made on a liquid medium on a reaction plate. The wells on the plate were filled with a liquid medium (nutrient broth, Biomaxima, Lublin, Poland), to which a bacterial suspension (0.5 on the McFarland scale) was applied, and then a controlled amount of GO, GO + C, GO + Ag and GO + HClO aerosol was applied using an automatic pipette. After 24 h of incubation (37 °C), PrestoBlue reagent (PrestoBlue™ Cell Viability Reagent, Thermo Fisher Scientific Invitrogen, Houston, TX, USA) was added, and after 30 min of incubation, fluorescence with an excitation wavelength of 560 nm and emission of 590 nm was measured. The results are presented as a viability % relative to the control.
- Replicator stamp analysis
  The study was conducted on the following strains: *Pseudomonas aeruginosa* (ATCC 27853) and *Staphylococcus aureus* (ATCC 25923). A suspension of bacteria with an optical density of 0.2 on the McFarland scale was prepared. Sterile filter paper (Filtrak, diameter 9 cm) was placed in an empty Petri dish and 1 mL of bacterial suspension was distributed. The tissue paper was left to dry under the laminar flow chamber for 30 min. Then, the surface of the paper discs was coated with an aerosol containing the following aerosols: GO, GO + C, GO + Ag, and GO + HClO. The tissue paper disc soaked in ultrapure water served as a control disc. The tissue paper discs were left to dry for 30 min and then they were pressed on an agar medium (nutrient agar, Biomaxima, Lublin, Poland). The dishes prepared this way were incubated for 24 h at a temperature of 37 °C. After incubation, the colonies formed were counted.

## 3. Results

### 3.1. Structural Research

**Figure 2:**
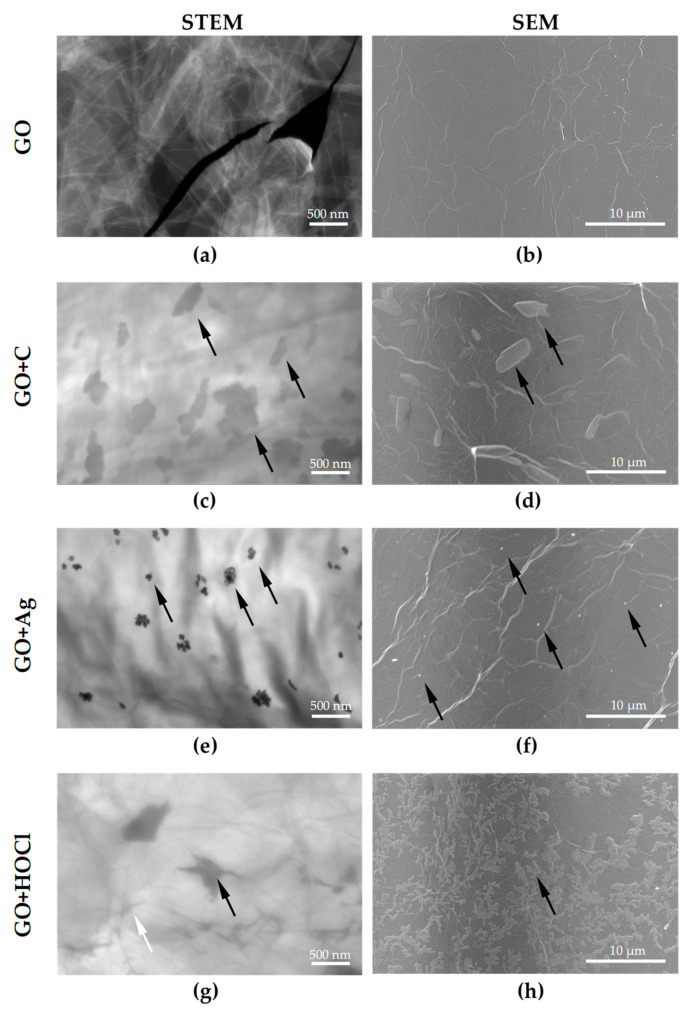
STEM (**a**,**c**,**e**,**g**) and SEM images (**b**,**d**,**f**,**h**) of GO (**a**,**b**), GO + C (**c**,**d**), GO + Ag (**e**,**f**), and GO + HClO (**g**,**h**).

In order to illustrate the structure of graphene oxide (GO) suspension (Figure 2a,b) as well as graphene oxide containing curcumin (GO + C) (Figure 2c,d), silver nanoparticles (GO + Ag) (Figure 2e,f) and hypochlorous acid (GO + HClO) (Figure 2g,h) applied in the form of an aerosol, transmission (STEM) (Figure 2a,c,e,g) and reflection (SEM) images (Figure 2b,d,f,h) using the Quanta FEG 250 microscope were recorded. 

The graphene oxide flakes arrange themselves quite evenly, creating a compact plane (Figure 2a,b). The introduction of additives in the form of curcumin (Figure 2c,d), silver nanoparticles (Figure 2e,f), or hypochlorous acid (Figure 2g,h) causes an increase in structural heterogeneity. One can clearly see silver nanoparticles (Figure 2c,d) and curcumin (Figure 2e,f) connected by adhesion forces to the graphene oxide flakes (Figure 2 arrows). Silver nanoparticles tend to form randomly distributed agglomerates and are embedded in a layer of graphene oxide. The addition of hypochlorous acid to the GO suspension applied in the form of an aerosol after application of the surface resulted in the formation of hypochlorous acid crystals HClO located on the surface of graphene oxide flakes (Figure 2g,h black arrows). In addition, morphological changes in the edges of graphene flakes were observed due to the action of hypochlorous acid (Figure 2g white arrow).

### 3.2. Infrared Spectroscopy

**Figure 3:**
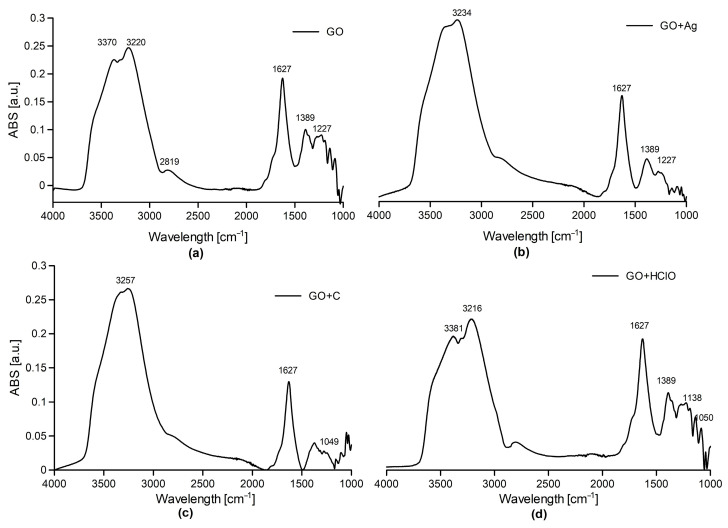
FTIR-ATR spectrum of GO (**a**), GO + C (**b**), GO + Ag (**c**), and GO + HClO (**d**).

FTIR-ATR analysis of dispersed aqueous graphene oxide (GO) suspension applied as an aerosol (Figure 3a) showed a visible wide bandwidth in the range of 3700–3000 cm^−1^ with maxima at 3370 cm^−1^ and 3220 cm^−1^, corresponding to the stretching vibrations of hydroxyl (–OH) groups. The band at 2819 cm^−1^ may be associated with vibrations C–H. A strong peak at 1627 cm^−1^ is characteristic of carbonyl groups (C=O), and smaller bands at 1389 cm^−1^ and 1227 cm^−1^ are attributed to epoxy (C–O–C) and hydroxyl (C–OH) groups.

The spectrum of the dispersed aqueous suspension of graphene oxide with curcumin, GO + C, (Figure 3b) applied in the form of aerosol, showed a band at 3234 cm^−1^ associated with hydroxyl groups (–OH), and a strong peak at 1628 cm^−1^ corresponding to the vibrations of carbonyl groups (C=O). A band at 1049 cm^−1^ is also visible, which may indicate the formation of C-O-C connections between curcumin and GO.

For the GO + Ag sample (with silver nanoparticles) we observe a similar spectrum as for pure GO, because metallic silver does not give an IR spectrum (Figure 3c).

For the GO + HClO sample (with hypochlorous acid) (Figure 3d), the bands at 3381 cm^−1^ and 3257 cm^−1^ correspond to the vibrations of hydroxyl groups (–OH), and a strong peak at 1632 cm^−1^ is characteristic of carbonyl groups (C=O). The band at 1389 cm^−1^ can be attributed to the vibrations of epoxy (C–O–C) and hydroxyl (C–OH) groups.

### 3.3. Laser Emission Spectroscopy (LIBS)

**Figure 4:**
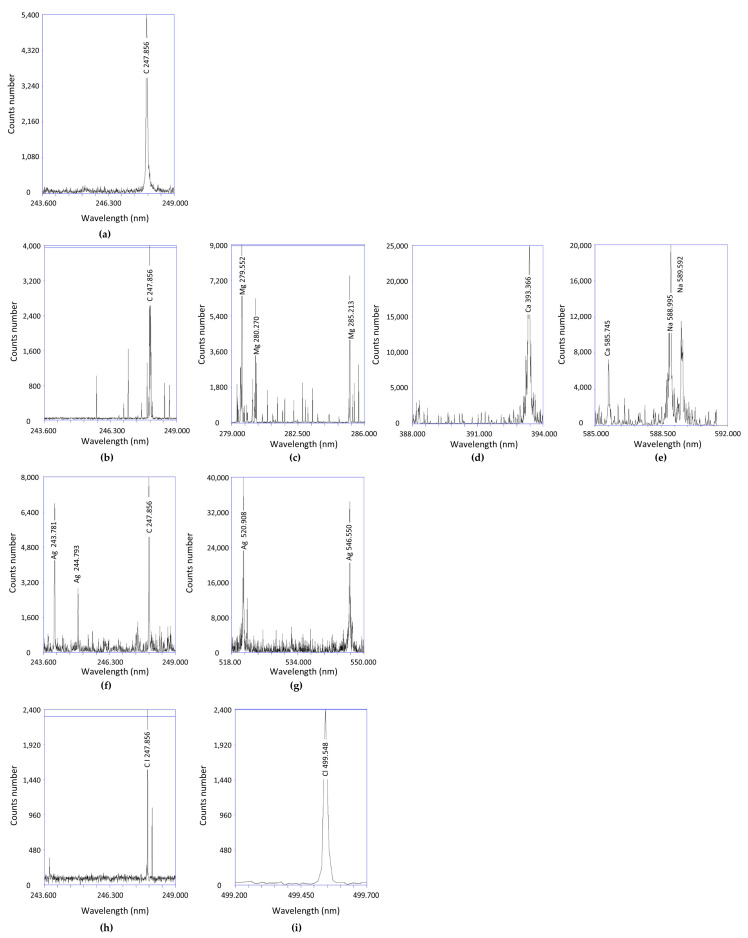
LIBS spectra: (**a**) GO, (**b**–**e**) GO + C, (**f**,**g**) GO + Ag, and (**h**,**i**) GO + HClO.

Laser emission spectroscopy allows the elemental composition of individual aerosols, i.e., GO (Figure 4a), GO + C (Figure 4b–e), GO + Ag (Figure 4f,g), and GO + HClO (Figure 4h,i) to be characterized. Repeatability of characteristic carbon peaks for the 247.856 nm wavelength was observed in each GO-containing aerosol. 

In the GO + C samples, the carbon peak at 247.856 nm (Figure 4b) originates not only from graphene oxide, but also from curcumin. Although the elements that build curcumin are predominantly C, H, and O, other elements have also been identified in GO + C samples, i.e., calcium (393.366 and 585.745 nm) (Figure 4d,e), magnesium (279.522, 280.270) and 285.213 nm) (Figure 4c), and sodium (588.592 and 588.985 nm) (Figure 4e), which may come from the manufacturing process.

The presence of silver was confirmed in a dispersed aqueous suspension of graphene oxide sample (GO + Ag) after its application in the form of an aerosol. The strongest peaks were observed at wavelengths of 520.908 and 546.550 nm (Figure 4g). In contrast, in GO + HClO samples, the presence of chlorine at a wavelength of 499.548 nm was confirmed.

### 3.4. Contact Angle

The wettability of the surface of laboratory slides with the GO, GO + C, GO + Ag, and GO + HClO aerosol previously applied (24 h) did not show any significant differences (Table 2). All droplets of ultrapure water dripped to the surface with a previously applied aerosol acted in the same manner and showed the hydrophilicity of the surface. The smallest contact angle of 45° was observed for surfaces with GO + HClO aerosol applied. This is due to the significant addition of hypochlorous acid (which consists of ultrapure water and 500 ppm HClO) to the dispersed graphene oxide slurry at a ratio of 50:50% (Table 1).

### 3.5. The ζ-Potential

The ζ-potential is an important indicator of the degree of repulsion between similarly charged particles in a dispersion. For a colloidal system to be considered stable, the ζ-potential typically needs to be greater than ±30 mV. Values around or above this threshold indicate sufficient electrostatic repulsion to prevent the aggregation or coagulation of particles, thereby ensuring a stable system.

In a study of various aerosols, the ζ-potential values for graphene oxide (GO), GO-Ag, GO-C, and GO-HClO were assessed. The results show good stability, with ζ-potential values ranging from −22.03 mV for GO-HClO to −27.7 mV for GO. For the aerosols of GO-Ag and GO-C, the ζ-potential values were −22.86 mV and −27.66 mV, respectively.

### 3.6. Bacterial Growth Inhibition Zone

**Figure 5:**
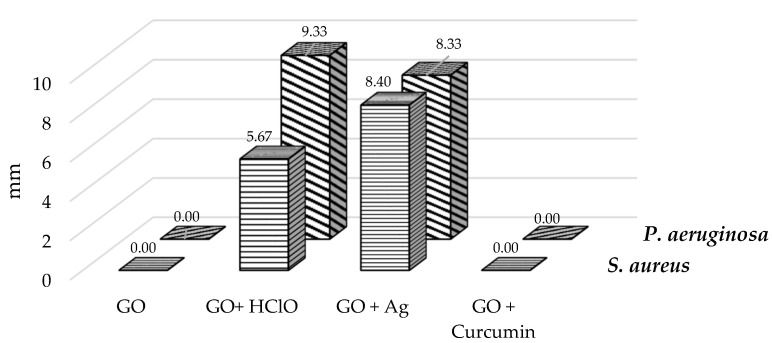
Bacterial growth inhibition zone for *Staphylococcus aureus* and *Pseudomonas aeruginosa* treated with GO, GO + C, GO + Ag, and GO + HClO. Results are given as values with the diameter of the sprayed aerosol disc (27 mm), values of 0.00 indicate the absence of any inhibition zone.

**Figure 6:**
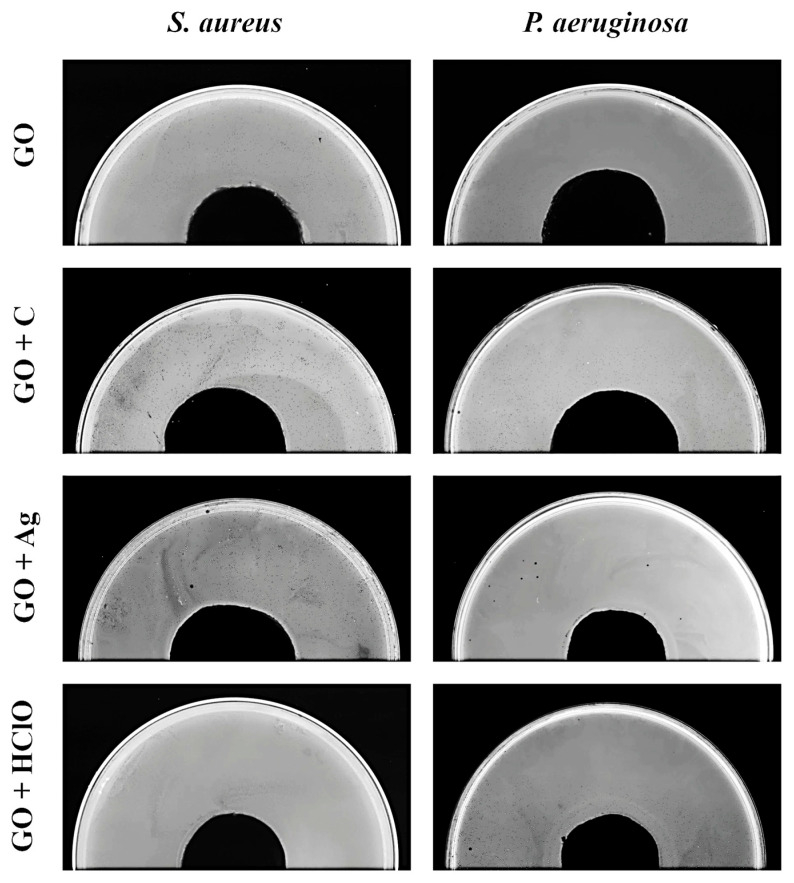
Exemplary pictures of growth inhibition zone of *S. aureus* and *P. aeruginosa* after exposure to aerosols GO, GO + C, GO + Ag, and GO + HClO.

The largest growth inhibition zone was observed for *Staphylococcus aureus* and *Pseudomonas aeruginosa* bacteria in case of an aerosol containing graphene oxide enriched with silver nanoparticles (GO + Ag) and hypochlorous acid (GO + HClO) (Figure 5 and Figure 6). A different trend was observed for aerosol containing graphene oxide GO and the combination of graphene oxide with curcumin, as they did not cause the formation of a growth inhibition zone.

### 3.7. Bacterial Viability

Table 3 presents the results of viability studies for *Staphylococcus aureus* and *Pseudomonas aeruginosa*. An aerosol containing graphene oxide (GO) served as the control sample. Doping graphene oxide with silver nanoparticles administered as an aerosol (GO + Ag) resulted in the viability of *Staphylococcus aureus* and *Pseudomonas aeruginosa* decreasing to 12.24% and 53.29%, respectively. The effect of an aerosol containing graphene oxide with hypochlorous acid (GO + HClO) on the tested bacterial strains also reduced the viability of *Staphylococcus aureus* to 66.11% and *Pseudomonas aeruginosa* to 76.61%. This is probably due to the strong oxidative properties of the acid, which destroys bacterial cell walls. 

It is interesting to note that when graphene oxide was combined with curcumin (GO + C), the viability of *Pseudomonas aeruginosa* decreased to 95.06%, while the viability of *Staphylococcus aureus* strains increased by 30.78%, which confirms the literature reports about its unpredictable nature.

### 3.8. Replicator Stamp Analysis

**Figure 7:**
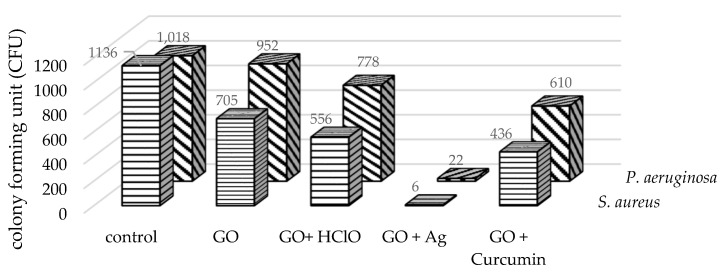
Colony-forming units of *Staphylococcus aureus* and *Pseudomonas aeruginosa* after the use of paper disc impregnated with GO, GO + C, GO + Ag, and GO + HClO aerosol.

Figure 7 presents the results of replicator stamp analysis of the paper disc soaked in GO, GO + C, GO + Ag, and GO + HClO aerosol on the colony-forming units of *Staphylococcus aureus* and *Pseudomonas aeruginosa* bacteria.

The effect of an aerosol containing graphene oxide (GO) suspension was characterized by a reduction in the number of colonies (CFU) of *Staphylococcus aureus* and *Pseudomonas aeruginosa* bacteria compared to the control. The control was paper disc soaked in ultrapure water. The use of paper disc impregnated with an aerosol of graphene oxide with hypochlorous acid (GO + HClO) showed a significant decrease in the number of colonies in all replications of the experiment, suggesting that hypochlorous acid enhances the antibacterial effect of graphene oxide. 

It is interesting to note that, only in the imprint studies of the paper disc soaked in an aerosol containing a suspension of GO and curcumin, a significant reduction in the number of colonies of *Staphylococcus aureus* and *Pseudomonas aeruginosa* bacteria of 40.1 and 61.6%, respectively, was observed. In viability studies, this effect was not observed. This indicates that curcumin supports the antibacterial properties of graphene oxide, but in different types of experiments, its antibacterial effect may not depend on its chemical properties, but on the degree and nature of its physical effects on bacteria. 

The largest decrease in the number of colonies of *Staphylococcus aureus* and *Pseudomonas aeruginosa* bacteria was recorded for the paper disc soaked in an aerosol containing graphene oxide and silver nanoparticles (GO + Ag) of 99.4 and 97.8%, respectively. 

## 4. Discussion

Microscopic studies have shown that the graphene oxide suspension applied in the form of an aerosol (GO), together with silver nanoparticles (GO + Ag), curcumin (GO + C) and hypochlorous acid (GO + HClO) nanoparticles, have a different structure and properties.

It should be noted that the morphology of graphene oxide flakes after aerosol application did not change significantly. Using SEM microscopy, the layered arrangement of graphene and the random distribution of the additives used in experiment were observed, which was particularly evident in STEM transmission images. FTIR spectroscopy identified characteristic functional groups in graphene aerosols. LIBS analysis confirmed the presence of the main elements in the tested samples. 

All ultrapure water droplets fallen on the surface previously applied with GO, GO + C, GO + Ag, and GO + HClO were hydrophilic.

Studies have shown that graphene oxide has bacteriostatic properties, which has also been confirmed in publications [16,17]. 

In the demonstrated results of GO tests, the scanning electron microscopy (SEM) images reveal that GO-exposed bacterial cells displayed significant envelope disruption, often enveloped uniformly by GO layers. To elucidate the mechanism behind the antibacterial effects of GO, the role of sharp nano edges of single flakes was also investigated as well as the impact of lateral dimensions. It was observed that a steady decrease in bacterial viability occurred as the lateral size of GO sheets diminished [35]. The obtained results suggest that GO sheets isolate bacterial cells, restricting their growth through a bidirectional mechanism by physical isolation and blocking membrane transport. Another study indicated that GO sheets might intertwine, wrap around, puncture, and ultimately destabilize the cellular envelopes of bacterial cells [36]. Overall, the antimicrobial mechanism of GO likely involves a combination of several factors, including physical puncturing, oxidative stress-induced damage to cellular and membrane components, wrapping-induced blockage of membrane transport and/or growth inhibition, and membrane destabilization through the insertion and destructive extraction of membrane components. Depending on experimental conditions, these combined mechanisms may result in total destruction of the cellular envelope (bactericidal effect) or growth inhibition (bacteriostatic effect). A critical factor influencing the interplay of these mechanisms is the functional group profile of GO, which presumably affects its oxidative stress generation capabilities and interactions with microbial surfaces. In contrast to GO, curcumin is a naturally occurring organic compound abundantly found in *Curcuma longa* L., commonly known as turmeric. Several mechanisms explain its antimicrobial action. One involves disrupting the assembly of FtsZ protein during bacterial cell division, thereby inhibiting cell proliferation. In this context, FtsZ protein performs a role akin to that of the tubulin protein in eukaryotic cells. By GO coating, the antibacterial activity of GO-C was increased compared to the GO aerosol. These findings are similar to the higher antibacterial effect against *E. coli* than GO published by Nguyen et al. [37]. Moreover, the bactericidal effect of the aerosol containing graphene oxide suspension increases after adding silver particles and hypochlorous acid. To assess the antibacterial activity of synthesized GO-based aerosol against Gram-negative and Gram-positive bacterial cells, the bacterial strains of *Escherichia coli* and *Staphylococcus aureus* were used. The obtained results show that the aerosol of GO-Ag presents the strongest antibacterial activity. Silver nanoparticles entrapped on the surface of GO flakes released the Ag+ ions and were resealed in the form of nanoparticles to effectively react with the surface of bacterial cells and cause lethal cell damage. Previously published data [38] also reveal that the GO-Ag, but in the form of aerosol, caused significantly increased bacterial cell toxicity compared to Ag nanoparticles and GO hydrocolloids. In our investigation, the novel application was verified, and the antibacterial activity of GO-Ag was confirmed. The differences observed in the effects of substances on Gram-positive and Gram-negative bacterial cells are due to their structure. Gram-positive cells have a thick cell wall, while Gram-negative cells are much thinner, with an outer membrane present. Although Gram-positive bacteria are thought to be less susceptible to silver nanoparticles due to the presence of a thick cell wall [39] when combined with other substances, they can exhibit stronger antibacterial properties. In the study conducted, based on the results obtained from the viability and colony-forming units, it seems that the carrier graphene oxide fulfilled its function, contributing to the greater toxicity induced by silver nanoparticles even in relation to Gram-positive bacteria, which was *S. aureus*. Many physiochemical features, including pH, concentration, treatment time, and volume, influence the efficacy of the HOCl solution against microorganisms [40]. The GO was used as a stabilizer agent to overcome the concentration-dependent effect of HOCl on the cytotoxicity and antibacterial activity. As a result of the impact of an aerosol containing GO-HOCl, we observed a significant decrease in cell viability of Gram-positive and Gram-negative bacteria, which indicates that the effect was cell-type independent. The HClO reacts with different molecules, such as lipids, nucleotides, and proteins of microorganisms [30]. Although the exact mechanism of action of GO-HClO against microorganisms is not yet fully understood, it is clear that it involves several key factors. It induces protein aggregation, reduces cellular ATP levels, and inactivates essential bacterial chaperones released by microorganisms in response to stress. The aerosolization of GO-HClO can result in a prolonged interaction with microorganisms after the acid stabilizes on the surface of GO, potentially reducing the limitation of the required dosage.

## 5. Conclusions

As a result of the impact of an aerosol containing graphene oxide (GO) and graphene oxide doped with silver nanoparticles (GO + Ag), hypochlorous acid (GO + HClO), and curcumin (GO + C) on selected structural and biological properties, it has been shown that graphene oxide can provide a platform for the release of substances with bactericidal activity, while GO it itself is characterized by bacteriostatic effects on selected bacteria *Staphylococcus aureus* and *Pseudomonas aeruginosa.* The use of different GO enrichment additives produces different biocidal results, and it is not always possible to predict the direction and degree of these changes.

## 6. Patents

P.450037—Graphene oxide suspension for aerosol administration and its application.

## Figures and Tables

**Table 1:** Mixtures of tested substances prepared for application in the form of an aerosol.

Lp.	Substance	Sample Name	Proportion Used
1	Dispersed graphene oxide suspension with a concentration of 4.5 g/L	GO	100%
2	Dispersed graphene oxide suspension with a concentration of 4.5 g/L with the addition of curcumin	GO + C	Roztwór kurkuminy 0.1 g/L (5:1) (500 mL GO + 100 mL C)
3	Dispersed graphene oxide suspension with a concentration of 4.5 g/L with the addition of silver nanoparticles	GO + Ag	5:1 (500 mL GO + 100 mL AgNPs)
4	Dispersed graphene oxide suspension with a concentration of 4.5 g/L with the addition of hypochlorous acid 500 ppm	GO + HClO	50%:50%

**Table 2:** Contact angle measurements of GO, GO + C, GO + Ag, and GO + HClO.

	Contact Angle (°)	Standard Deviation
GO	51	2
GO + C	51	1
GO + Ag	48	2
GO + HClO	45	2

**Table 3:** Bacterial viability *Staphylococcus aureus* and *Pseudomonas aeruginosa*.

	*Staphylococcus aureus*		*Pseudomonas aeruginosa*	
	Viability (%)	Standard Deviation	Viability (%)	Standard Deviation
GO	100.00	0.11	100.00	0.02
GO + C	130.78	0.02	95.06	0.03
GO + Ag	12.24	0.01	53.29	0.02
GO + HClO	66.11	0.10	76.61	0.03

## Data Availability

Data are contained within the article.
